# Forced Trefoil Factor Family Peptide 3 (TFF3) Expression Reduces Growth, Viability, and Tumorigenicity of Human Retinoblastoma Cell Lines

**DOI:** 10.1371/journal.pone.0163025

**Published:** 2016-09-14

**Authors:** Jan Große-Kreul, Maike Busch, Claudia Winter, Stefanie Pikos, Harald Stephan, Nicole Dünker

**Affiliations:** 1 University of Duisburg-Essen, Medical Faculty, Institute of Anatomy II, Department of Neuroanatomy, Essen, Germany; 2 Division of Haematology and Oncology, Children’s Hospital, University of Duisburg-Essen, Essen, Germany; Institute of Biochemistry and Biotechnology, TAIWAN

## Abstract

Trefoil factor family (TFF) peptides have been shown to effect cell proliferation, apoptosis, migration and invasion of normal cells and various cancer cell lines. In the literature TFF peptides are controversially discussed as tumor suppressors and potential tumor progression factors. In the study presented, we investigated the effect of *TFF3* overexpression on growth, viability, migration and tumorigenicity of the human retinoblastoma cell lines Y-79, WERI-Rb1, RBL-13 and RBL-15. As revealed by WST-1 and TUNEL assays as well as DAPI and BrdU cell counts, recombinant human TFF3 significantly lowers retinoblastoma cell viability and increases apoptosis levels. Transient *TFF3* overexpression likewise significantly increases RB cell apoptosis. Stable, lentiviral *TFF3* overexpression lowers retinoblastoma cell viability, proliferation and growth and significantly increases cell death in retinoblastoma cells. Blockage experiments using a broad-spectrum caspase inhibitor and capase-3 immunocytochemistry revealed the involvement of caspases in general and of caspase-3 in particular in TFF3 induced apoptosis in retinoblastoma cell lines. Soft agarose and *in ovo* chicken chorioallantoic membrane (CAM) assays revealed that *TFF3* overexpression influences anchorage independent growth and significantly decreases the size of tumors forming from retinoblastoma cells. Our study demonstrates that forced *TFF3* expression exerts a significant pro-apoptotic, anti-proliferative, and tumor suppressive effect in retinoblastoma cells, setting a starting point for new additive chemotherapeutic approaches in the treatment of retinoblastoma.

## Introduction

Three trefoil factor family (TFF)-peptides have been characterized in mammals so far (reviewed in refs. [1–6]: TFF1—formerly pS2, TFF2—formerly spasmolytic polypeptide, and TFF3—previously called intestinal trefoil factor (ITF)). They are characterized by a trefoil domain, which has a P-motif, a three-looped structure held together by disulfide bonds [1], whereby TFF2 contains two trefoil domains and TFF1 and TFF3 only contain one trefoil domain [7].

Besides their expression in mucous epithelia, TFF peptides are synthesized in the central nervous system and ocular tissues of rodents and humans [8–10]. Our group was the first to investigate retinal expression of TFF peptides. Previous studies by our group revealed that only TFF3, but not TFF1 and TFF2 are expressed in the healthy human retina [11; 12], whereby retinoblastoma (RB) cell lines, established from malignant eye tumors of children, exhibit high levels of *TFF1* [11; 12], but only trace amounts of *TFF3* and no detectable *TFF2*. Most recently, we demonstrated that the expression of *TFF3* in retinoblastoma cell lines is regulated epigenetically [12].

In the literature TFF peptides are controversially discussed as tumor suppressors and potential tumor progression factors [4; 5; 13; 14]. *TFF3* overexpression is frequently observed in human cancers (reviewed in ref. [5]) and thus, was thought to induce cancer growth. Besides, *TFF3* expression correlates with the tumor grade in hepatocellular carcinoma [15], is highly expressed in intestinal metaplasia, and a marker for poor prognosis in gastric carcinoma [16].

In most systems studied so far, TFFs show protective, wound healing and anti-apoptotic effects. In the murine retina, by contrast, our group demonstrated that recombinant TFF2 exerts a strong pro-apoptotic and pro-proliferative effect [17]. Besides, *TFF3* overexpression significantly reduces colon carcinoma cell growth [18]. On the other hand, it has been reported that spontaneous apoptosis of enterocytes is increased in *Tff3* deficient mice and TFF3 mediates intestinal goblet cells resistance to anchorage-related and cytotoxic agent-induced apoptosis [19; 20]. The influence of TFF3 on retinoblastoma cell apoptosis, proliferation, growth and oncogenicity has, however, not been investigated so far.

Thus, in the present study we set out to determine the effects of (i) application of recombinant human TFF3, (ii) transient *TFF3* overexpression and (iii) stable, lentiviral *TFF3* overexpression on growth, viability, proliferation, apoptosis as well as anchorage-independent growth, migration and tumor formation capacity of different human retinoblastoma cell lines. We found forced *TFF3* expression to lower RB cell growth, viability, and tumorigenicity and to induce a significant increase in cell death levels of retinoblastoma cell lines.

## Material and Methods

### Human retina and retinoblastoma samples

Post mortem human retina samples from cornea donors, retinoblastoma samples and sections from enucleations were used for comparative TFF3 expression studies. The Ethics Committee of the Medical Faculty of the University of Duisburg-Essen approved the use of human retina (approval # 06–30214) and retinoblastoma samples (approval # 14-5836-BO) for research conducted in the course of the study presented and written informed consent has been obtained from patients`relatives or parents.

### Cell culture

The human retinoblastoma (RB) cell lines RBL-13 and RBL-15, established and first described by Griegel (1990) [21] and formerly donated by K. Heise, were kindly provided by Dr. H. Stephan. The RB cell lines Y-79 [22] and WERI-Rb1 [6], originally purchased from the Leibniz Institute DSMZ (German Collection of Microorganisms and Cell Cultures), were likewise kindly provided by Dr. H. Stephan. All RB cell lines were last tested and authenticated in September 2015. Mutation analyses were conducted using a MLPA Kit (MRC-Holland; Amsterdam; Salsa MLPA Kit P047 RB1) and reactions were performed according to the manufacturer´s instructions. Additional sequencing of the *RB1* gene was performed for all retinoblastoma cells lines. The cell lines were cultivated as suspension cultures in Dulbecco’s modified Eagle’s medium (DMEM; PAN-Biotech) with 15% fetal calf serum (FCS; PAN-Biotech), 100 U penicillin/ml and 100 μg streptomycin/ml (Invitrogen), 4 mM L-glutamine (Sigma), 50 μM ß-mercaptoethanol (Roth) and 10 μg insulin/ml (Sigma) at 37°C, 10% CO_2_ and 95% humidity. Human embryonic kidney cells were grown as adherent cell culture in DMEM with 10% FCS, 4 mM L-glutamine, 100 U penicillin / ml, and 100μg streptomycin/ml at 37°C, 5% CO_2_ and 95% humidity. No approval from an ethics committee was required for work with the human cell lines.

### Treatment with recombinant human TFF3

Serum starved (72 h) Y-79 RB cells were transferred to DMEM without insulin containing 5% FCS, 4 mM L-glutamine, antibiotics and 50μM 2-mercaptoethanol prior to stimulation. Cells were treated for 24 h to 72 h with 0.5 μg/ml– 5 μg/ml recombinant human TFF3 (rTFF3; #300–61; 13.2 kDa homodimer; PeproTech) dissolved in sterile water. The biological activity of rTFF3 was determined by the manufacturer, testing the ability to chemo-attract human MCF-7 cells using concentrations of 1 μg/ml—10 μg/ml rTFF3. Cells treated with equivalent amounts of solvent were used as controls.

### Plasmids and lentiviral expression vectors

For the pLVX-IRES-Neo_TFF3 plasmid, the human *TFF3* cDNA sequence was amplified from human stomach by RT-PCR using the forward primer 5’-ATATGAATTCGAGGCCTCCTGGACCATGAAG-3’ and reverse primer 5’-TCAGCTCGAGAGCTGGAGGTGCCTCAGAAG-3’ containing restriction sites (underlined) for *Eco*RI and *Xho*I. The purified PCR product was digested with *Eco*RI and *Xho*I and ligated into the pLVX-IRES-Neo vector (clontech). In order to generate an expression vector with an ubiquitin C promoter (pCS2+_UBQ), we amplified the human ubiquitin C gene from genomic human retina DNA in a two-step PCR. In a first PCR reaction the 5’-region of the ubiquitin C gene was amplified with the forward primer 5’-ATATGTCGACGGCCTCCGCGCCGGGTTTTGG-3’ and reverse primer 5’-CACTGGGCTCAACCTCGAGGG-3’ using Amplitaq-Gold polymerase (Invitrogen). The corresponding PCR product was gel-purified and used in a second PCR reaction with the same forward primer and a nested reverse primer 5’-ATATGGATCCGTCTAACAAAAAAGCCAAAAACGGC-3’ containing restriction sites (underlined) for *Sal*I and *Bam*HI to amplify the 5’-flanking sequence from positions -1225 to -6 [23]. Using *Sal*I and *Bam*HI, the CMV promoter was cut from the expression vector pCS2+ (kindly provided by A. Hecht) and replaced by the *Sal*I/*Bam*HI digested ubiquitin C promoter region. For the generation of a *TFF3* expression vector under the control of the ubiquitin C promoter (pCS2+_UBQ_TFF3), the human *TFF3* cDNA sequence was amplified from the pLVX-IRES-Neo_TFF3 plasmid by PCR using the same primers listed above. The purified PCR product was digested with *Eco*RI and *Xho*I and ligated into the pCS2+_UBQ vector. To generate a lentiviral *TFF3* expression vector (pLenti CMV_TFF3), the human TFF3 cDNA sequence was cut from the pLVX-IRES-Neo_TFF3 vector by *Eco*RI and *Xho*I and ligated into the pENTR4 vector, digested with the same restriction enzymes. *TFF3* was finally cloned into the pLenti CMV Puro Dest vector by the use of a Gateway LR Clonase II Enzyme Mix (Invitrogen), following the manufacturer`s protocol.

### Transient transfection of retinoblastoma cells

For transient *TFF3* overexpression, 5 x 10^5^ RB cells were transfected with 4 μg plasmid DNA in a ratio of 1:4 using the FuGENE^®^ HD Transfection Reagent (Promega) according to the manufacturer´s instructions. The TFF3 expression plasmid (pCS2+_UBQ_TFF3) as well as an empty control vector (pCS2+_UBQ) were used for transfection. Cells were also transfected with a GFP expression vector (pCS2+_GFP; kindly provided by Dr. A. Weise) as a control for transfection efficiency.

### Generation of lentiviral particles and transduction of retinoblastoma cells

In order to be able to visualize the human RB cells in chicken chorioallantoic membrane (CAM) assays (see below), for stable *TFF3* overexpression, GFP-labelled RB cells were used, generated as described previously [24]. To produce lentiviral particles, 6 x 10^6^ HEK293-T cells were transfected with 6 μg of each of the following plasmid DNAs: the packaging vector pczVSV-G [25], the pCD NL-BH vector [25] and the TFF3 expressing vector pLenti CMV_TFF3 in the presence of 45 μg polyethyleneimine (PEI, branched; Aldrich). Control particles were generated using the pPRIME-CMV-Neo-FF3 cloning vector [26] instead of the *TFF3* expressing vector. After 24 h the transfection medium was substituted for Iscove`s modified Dulbecco´s medium (IMDM; Gibco) supplemented with 10% FCS and 1% penicillin/streptomycin. Viral supernatants were harvested, filtered (0.45 μm pores) and cryopreserved 48 h after transfection. For stable, lentiviral *TFF3* overexpression, 20 x 10^6^ GFP-labelled RB cells were infected with *TFF3*-coding lentiviral particles or control lentiviral particles in the presence of 10 μg/ml hexadimethrine bromide (polybrene; Sigma). After 24 h DMEM with supplements (twice the volume of the virus) was added and after another 48 h the medium was changed completely. RNA was isolated six days after transduction and *TFF3* overexpression was determined by quantitative Real-time PCR.

### Cell proliferation and apoptosis detection

Cell proliferation was determined by 5-Bromo-2´-deoxyuridine (BrdU; Sigma) incorporation. For BrdU immunocytochemistry 10 μM BrdU was added to the cells 4 h prior to PFA fixation. Cells were incubated with a rat anti-BrdU antibody (1:1,000; ab6326; Abcam) and proliferating cells were visualized using a goat anti-rat antibody labelled with Alexa Flour^®^ 594 (1:1,000; Molecular Probes). In order to determine changes in apoptosis levels, cells were stained with 4',6-Diamidino-2-phenylindole (DAPI; Sigma) or Apo-BrdU TUNEL assay (Life Technologies), following the manufacturer`s protocol and pycnotic nuclei were counted manually as described previously by our group [27].

### Caspase dependent apoptosis

In order to inhibit endogenous caspase activity, the broad-spectrum caspase inhibitor Boc-D-fmk (Calbiochem) was used. Stably TFF3 overexpressing RB cells were seeded on poly-D-lysine coated coverslips as described before and cultured in the presence of 50 μM Boc-D-fmk for 24 h. Afterwards, cells were fixed with 4% PFA and DAPI-stained to determine the number of pycnotic nuclei.

### WST-1 assay

The water soluble tetrazolium (WST) assay is based on the cleavage of the tetrazolium salt WST-1 to formazan by cellular mitochondrial dehydrogenases. Expansion in the number of viable cells results in an increase in the overall activity of the mitochondrial dehydrogenases in the sample. To determine RB cell viability, 4 x 10^4^ cells in 100 μl medium (i) treated with rising concentrations of rTFF3 or (ii) stably transduced with *TFF3* including the appropriate controls were seeded in two triplicates in a 96-well plate. Afterwards, 10 μl of a WST-1 solution was added to each well and cells were incubated at 37°C. At different time points the amount of formazan produced by viable cells was quantified by measuring the absorbance at 450 nm in a microplate reader (Biotec).

### Growth kinetic

To determine growth kinetics, 3 x 10^5^ RB cells with stable *TFF3* overexpression were seeded in 500 μl DMEM with supplements in a 24-well plate and vital cells were counted manually using the trypan blue exclusion method. Cells were seeded in triplicates and counted at several time points (24 h, 72 h, 96 h, 144 h and 216 h) over a period of nine days.

### Colony formation assay

Soft agarose assays were performed as described in detail previously [24]. The colony formation efficiency (%) per visual field was determined by counting the colonies and single cells in five visual fields (10 x) per cell line in triplicates and photographs were taken using a Leica DMIL microscope equipped with a digital camera (Jenoptik) and ProgRes Capture Basic 1.2.01 software.

### CAM assays

In order to test for changes in the migration and tumor formation capacity following *TFF3* overexpression, RB cells were grafted on the chick chorioallantoic membrane (CAM) or injected into the allantoic vein of chicken embryos as described in a recent publication by our group [24]. Mainly following the spontaneous and experimental metastasis model protocol published by Zijlstra et al. (2002) [28] and visualized by Palmer et al. (2011) [29], 50 μl cell suspension (1 x 10^6^ *TFF3* overexpressing, GFP-labelled or control cells in PBS) was grafted onto the CAM area. To boost the effect of stable *TFF*3 overexpression in the grafted cells, every second day 50 μl supernatant from Y-79 RB cell cultures transiently overexpressing TFF3 was applied to the graft area. Alternatively, 50 μl cell suspensions (1.5 x 10^5^ *TFF3* overexpressing or control cells in DMEM) were injected into the allantoic vein of chick embryos. For each RB cell line 30 eggs were grafted and intravenously injected in at least 3 independent experiments. All chick CAM experiments have been conducted according to relevant national guidelines of the responsible authority, the State Office for Nature, Environment and Consumer Protection (LANUV). The Institutional Animal Care and Use Committee (IACUC) of the Medical Faculty of the University Hospital Essen approved the CAM assays and no ethical approval was required as according to the German Animal Experiment and Welfare Guidelines, ethics approval is only essential if animals are intended to live beyond hatching.

### Harvesting of tissue

The duration of the CAM assay is limited to a 7–9 day window prior to chick`s hatching. Seven days after grafting (E10-17) chick embryos were anaesthetized by cooling on ice and sacrificed by decapitation. CAM tumors were excised, measured, photographed and either shock frozen for Real-time-PCR or fixed for 1 h at 4°C in 4% paraformaldehyde (PFA; Sigma-Aldrich) in 0.1 M phosphate buffer (pH 7.4). For cryo-embedding, the tumor tissue was incubated for 30 min in PBS (pH 7.3) containing 15% sucrose, followed by 30 min incubation in PBS containing 30% sucrose and finally embedded in OCT compound (Tissue-Tek) and sectioned at 10 μm using a cryostat. Six days after injection (E12-E18), 6 standardized (1.4 cm internal diameter) tissue punches of each ventral “lower CAM” were collected and transferred into a 24-well plate filled with PBS. Each of the lower CAM punches was scanned for GFP-positive RB cells and pictures were captured with an Axiovert 200M microscope, equipped with a CoolSNAP_cf_ camera (Proper Scientific Photometrics) and Micro-Manager 1.4 software (kindly provided by the group of Prof. Fandrey). CAM punches were combined and cryopreserved for RNA extraction [24]. Photos and measurements of tumors forming on the upper CAM were taken with a NIKON stereo dissecting microscope SMZ 1000 equipped with a NIKON digital camera and NIKON Eclipsenet software. Images were adjusted for brightness and contrast using Adobe Photoshop (San Jose, CA).

### RNA isolation, quantitative Real-time PCR and quantitative CAM assay analyses

RNA isolation from paraffin section of healthy human retina and retinoblastoma samples were performed using a miRNeasy FFPE kit (Qiagen) following the manufacturer´s protocol. RNA isolation from CAM tissue punches was performed as described previously [24]. Quantitative Real-time PCR analyses were performed and quantified following the protocol published previously [24] and the following human Taqman Gene Expression Assays (Applied Biosystems) were used: *TFF3* (Hs00902278_m1), *GAPDH* (Hs99999905_m1) and *18S* (Hs99999901_s1). In brief, to create specific standard curves for quantitative CAM assay analyses, increasing RNA concentrations of human RB cells of each cell line were mixed with chick RNA from CAM tissue at proportions of 0.01%, 0.05%, 0.1%, 1%, 5%, 10% and 20%. In order to correlate the h*GAPDH* expression—and thereby the amount of human tumor cell extravasation—with the amount of amplified cDNA, *18S* RNA, equally expressed in human and chick tissue was used as an internal control. Human *GAPDH* expression was normalized against the *18S* internal control to calculate a relative amount of human RNA, which was compared with the RB cell line specific standard curve in order to determine the amount of extravasated RB cells in ng.

### Immunocytochemistry

To immunocytochemically localize TFF3 in Rb cells, 1 x 10^5^ cells were seeded on poly-D-lysine (Sigma) coated coverslips. Cells were fixed with 4% PFA for 1 h at 4°C, permeabilized with 100% methanol for 5 min on ice and blocked with PBS containing 0.3% Triton^™^ X-100 (Sigma), 4% bovine serum albumin (BSA; Roth) and 5% normal goat serum (NGS; Dako) for 1 h at room temperature. Afterwards cells were incubated with a rabbit monoclonal TFF3 antibody (# TA307376, clone EPR3974; Origene) diluted 1:400 in PBS with 0.1% Triton^™^ X-100, 4% BSA and 1% NGS for 2 h at room temperature. A goat anti-rabbit antibody labelled with Alexa Flour^®^ 488 (Molecular Probes) diluted 1:1,000 in PBS with 1% BSA was used to visualize the reaction.

For TFF3 immunolocalization in formalin fixed healthy human retina and retinoblastoma paraffin sections were deparaffinised, and microwaved for 5 min at 600 W in citrate buffer (pH 6) to improve antigen retrieval. Sections were preincubated for 5 min with 3%H_2_O_2_ in methanol to quench endogenous peroxidase activity, rinsed 3 times in PBS and incubated for 20 min at room temperature with normal blocking serum provided in the Vectastain Elite ABC kit (Alexis). Immunostaining was performed using a rabbit monoclonal antibody against TFF3 (Origene; # TA307376; clone EPR3974) at a dilution of 1:250 in antibody dilution buffer provided in the Vectastain kit at 4°C overnight. After three rinses in PBS, sections were incubated with the biotinylated secondary antibody provided in the Vectastain kit for 30 min at room temperature, washed in PBS and incubated for 30 min with the Vectastain Elite ABC reagent. The reaction was visualized by DAB staining and the sections were counterstained with haematoxylin.

Active caspase-3 was detected immunocytochemically incubating cells with a rabbit monoclonal cleaved caspase-3 antibody (#9664; 5A1E; Cell Signalling) diluted 1:400 in PBS with 0.1% Triton^™^ X-100, 4% BSA and 1% NGS and cells at 4°C overnight. Cells were visualized using a goat anti-rabbit antibody labelled with Alexa Flour^®^ 594 (Molecular Probes), diluted 1:1,000 in PBS with 1% BSA. The localization of the tumors forming in the upper CAM after grafting was visualized on hematoxylin and eosin (H&E) stained cryosections. The human nature of the tumors forming in the chicken CAM after grafting human RB cells was verified using a mouse anti-human nuclei antibody (MAB128; Merck Millipore) at a dilution of 1:100 in PBS containing 0.1% triton, 4% BSA and 1% NGS overnight at 4°C. The reaction was visualized using a goat anti-mouse antibody labelled with Alexa Flour^®^ 594 (Molecular Probes), diluted 1:1,000 in PBS with 1% BSA for 2 h at room temperature. As controls, in all cases PBS was substituted for the primary antisera in order to test for non-specific labeling. No specific cellular staining was observed when the primary antiserum was omitted. Images were acquired using a NIKON Eclipse E600 microscope equipped with a digital camera and NIKON Eclipse net software.

### Western blot analysis

Total protein was isolated from up to 5 x 10^6^ cells, after washing with cold PBS, and cells were lysed for 60 min at 4°C in 500 μl RIPA buffer plus additives (see: [11]). Cell lysates were cleared by a 10 min centrifugation step at 14,000 rpm and 4°C. Protein content was determined photometrically using a BCA Protein Assay Kit (Pierce; Thermo Scientific). For Western blotting protein extracts were denatured with Laemmli buffer for 5 min at 95°C and 100 μg proteins or 30 μl supernatant were separated on a 12% SDS-PAGE. Proteins were transferred onto nitrocellulose membranes using a semi dry blotting chamber (Biorad). Membranes were blocked in tris buffered saline with 1% Tween-20 (TBS-T; pH 7.6) containing 5% skim milk powder for 1 h and then incubated with a TFF3 primary antibody (# TA307376, clone EPR3974; Origene) diluted 1:500 in TBS-T with 0.025% BSA and 0.02% NaN_3_ gently shaking overnight at 4°C. A horseradish peroxidase (HRP)-conjugated goat-anti-rabbit antibody (DAKO), diluted 1:10,000 in TBS-T with 2% skim milk powder, was used to visualize the reaction. Signals were developed by the Western Lightning^®^ Chemiluminescence Reagent Plus-ECL (Perkin Elmer) and visualized using a luminescent image analyzer LAS-3000 mini (Fujifilm) with ImageReader LAS-3000 software. To verify equal protein loading, blots were re-incubated with a polyclonal β-actin antibody (#4967; Cell Signalling) diluted 1:1,000 in TBS-T with 5% BSA.

### Statistical analysis

All assays were performed at least in triplicate. Statistical analyses were performed using GraphPad Prism 6. Data represent means ± SEM of two to five independent experiments from independent RB cell cultures. Results were analyzed by a Student`s *t*-test or one-way ANOVA and Newman-Keuls Post test and considered significantly different if *P < 0.05, **P < 0.01 or ***P < 0.001. Statistics on the growth curves was performed using a free web interface http://bioinf.wehi.edu.au/software/compareCurves/, which uses the compareGrowthCurves-function from a statistical modeling package called `statmod`, available from the R Project for Statistical Computing: http://www.r-project.org, previously described elsewhere [30].

## Results

### Recombinant human TFF3 significantly lowers Y-79 cell viability

**Fig 1:**
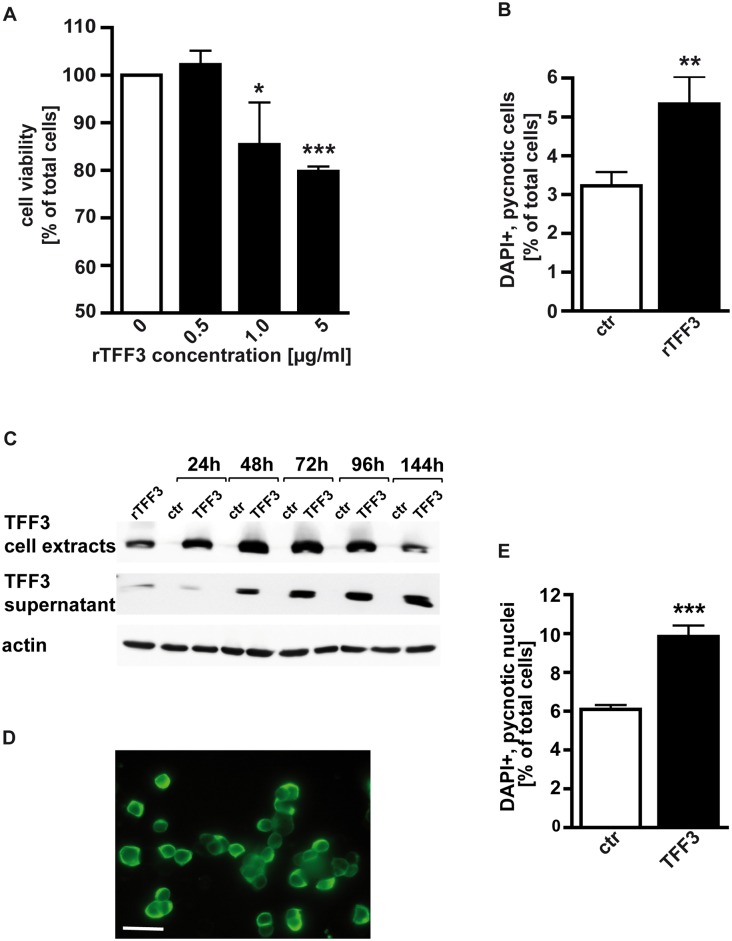
Effect of recombinant human TFF3 treatment (A,B) and transient TFF3 overexpression (C-E) on cell viability, cell proliferation and apoptosis levels of Y-79 retinoblastoma cells. (A) As revealed by WST-1 assay recombinant TFF3 (rTFF3) significantly lowered the number of viable Y-79 cells. Untreated control cells (0 μg/ml rTFF3) were set as 100%. (B) DAPI cell counts revealed that compared to control cells (ctr) apoptosis levels of rTFF3 (5μg/ml) treated cells were significantly higher. (C) Representative time course of increasing TFF3 protein levels in cell extracts and cell culture supernatants after transient TFF3 overexpression in Y-79 RB cells as revealed by Western blot. Cells transfected with the empty pCS2+_UBQ vector were used as controls and rTFF3 as a reference. (D) Immunocytochemical confirmation of TFF3 overexpression in Y-79 cells. scale bar in E: 20 μm. (E) DAPI cell counts revealing that transient TFF3 overexpression significantly increased the number of pycnotic nuclei. Values are means from up to five independent experiments ± SEM. *P < 0.05; **P < 0.01; ***P < 0.001 statistical differences compared to the control group calculated by Student`s *t*-test.

First, we tested the effect of recombinant human TFF3 (rTFF3) on Y-79 retinoblastoma cell viability. As detected by WST-1 assays, the addition of rTFF3 to the culture medium and re-stimulation after 48 h significantly lowered the number of viable Y-79 cells after 72 h (Fig 1A). The effect was dose-dependent and already observed with 1μg/ml rTFF3, but the highest significant decrease in viable cells (20%) was achieved with 5μg/ml rTFF3. Higher concentrations of rTFF3 (10 μg/ml) did not further increase this effect (data not shown).

### Recombinant human TFF3 significantly increases apoptosis levels, but does not influence Y-79 cell proliferation

In order to distinguish between TFF3 mediated cell death versus cell proliferation effects, we performed DAPI and BrdU cell counts. Stimulation of Y-79 RB cells with 5 μg/ml rTFF3 significantly increased the number of DAPI-positive, pycnotic nuclei after 24 h (Fig 1B), but had no discernible effect on the number of BrdU-positive, proliferating Y-79 cells (data not shown).

### Transient *TFF3* overexpression significantly increases Y-79 cell apoptosis

Transient TFF3 overexpression in Y-79 cells—used to substantiate our data gained with recombinant TFF3 treatment—was confirmed over a time period of six days by Real-time PCR (data not shown), Western blot analyses of cell extracts and cell culture supernatants (Fig 1C), and immunocytochemically (Fig 1D). Compared to control cells, transfected with the empty pCS2+_UBQ vector, a maximal 678–fold *TFF3* overexpression was achieved 24 h after transient transfection of Y-79 RB cells. *TFF3* mRNA levels slowly, but continually decreased, but were still high 144 h upon transfection (data not shown). TFF3 protein content concomitantly increased in Y-79 cell extracts, whereby highest levels were reached after 48 h– 72 h, accompanied by an accumulation of secreted TFF3 in the supernatant (Fig 1C). Y-79 RB cells endogenously express only trace amounts of *TFF3* mRNA and TFF3 protein levels are below the immunocytochemical detection limit. We were, however, able to detect TFF3 immunocytochemically in the cytoplasm of TFF3 overexpressing Y-79 cells 48 h after transfection (Fig 1D). Functional studies revealed that transient *TFF3* overexpression in Y-79 cells significantly increased the number of DAPI-positive, pycnotic nuclei 48 h after transfection (Fig 1E), but had no discernible effect on the number of BrdU-positive, proliferating cells (data not shown).

### Stable *TFF3* overexpression lowers viability, proliferation and growth of retinoblastoma cell lines

**Fig 2:**
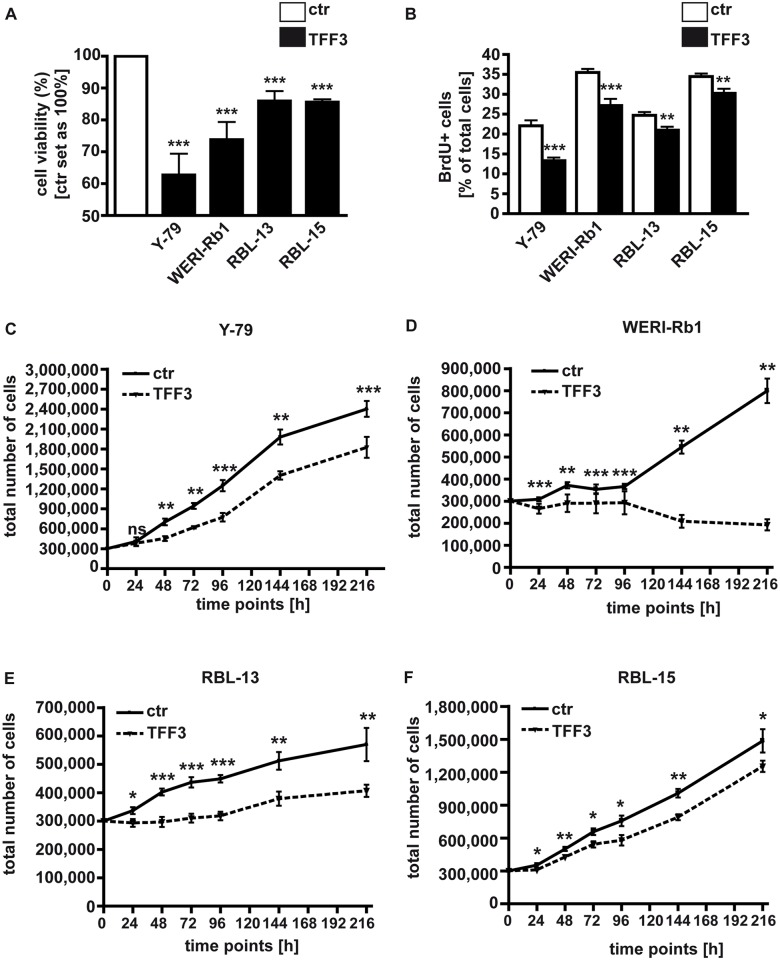
Effects of stable, lentiviral *TFF3* overexpression on RB cell viability, proliferation and growth. WST-1 assays (A), BrdU stains (B) and growth curves of *TFF3* overexpressing (TFF3) Y-79 (C), WERI-Rb1 (D), RBL-13 (E) and RBL-15 (F) versus control cells (ctr) transduced with lentiviral control particles revealed that forced *TFF3* expression leads to a significant reduction in cell viability, proliferation and cell growth rates, the latter with an overall P < 0.001. Values are means of at least 3 independent experiments ± SEM. **P < 0.01; ***P < 0.001; ns = no statistical differences compared to the control group calculated by Student`s *t*-test.

A stable, lentiviral *TFF3* overexpression approach was employed to further back-up our data and follow up on long-term effects on proliferation, migration capacity and tumorigenicity in soft agarose and CAM assays. Stable overexpression was confirmed by Real-time PCR (Fig A in S1 Fig). As revealed by WST-1 assays, forced *TFF3* expression results in a significant reduction in Y-79 cell viability (Fig 2A). This effect of forced *TFF3* expression could be verified and generalized in (i) the equally well-established RB cell lines WERI-Rb1 [6], derived from an unilateral RB tumor, (ii) RBL-13 cells, likewise derived from an unilateral RB tumor, but exhibiting different growth kinetics and tumor formation capacities [21; 24] and (iii) RBL-15 cells derived from a bilateral RB tumor [21] (Fig 2A). Besides, cell proliferation was significantly decreased in all four *TFF3* overexpressing cell lines analyzed as reflected by significantly lower numbers of BrdU-positive cells (Fig 2B). Moreover, all four RB cell lines analyzed after stable transduction with *TFF3*-coding lentiviral particles exhibited significantly lower growth rates compared to their controls (Fig 2C–2F).

### Forced *TFF3* expression significantly increases caspase-mediated cell death in retinoblastoma cells

**Fig 3:**
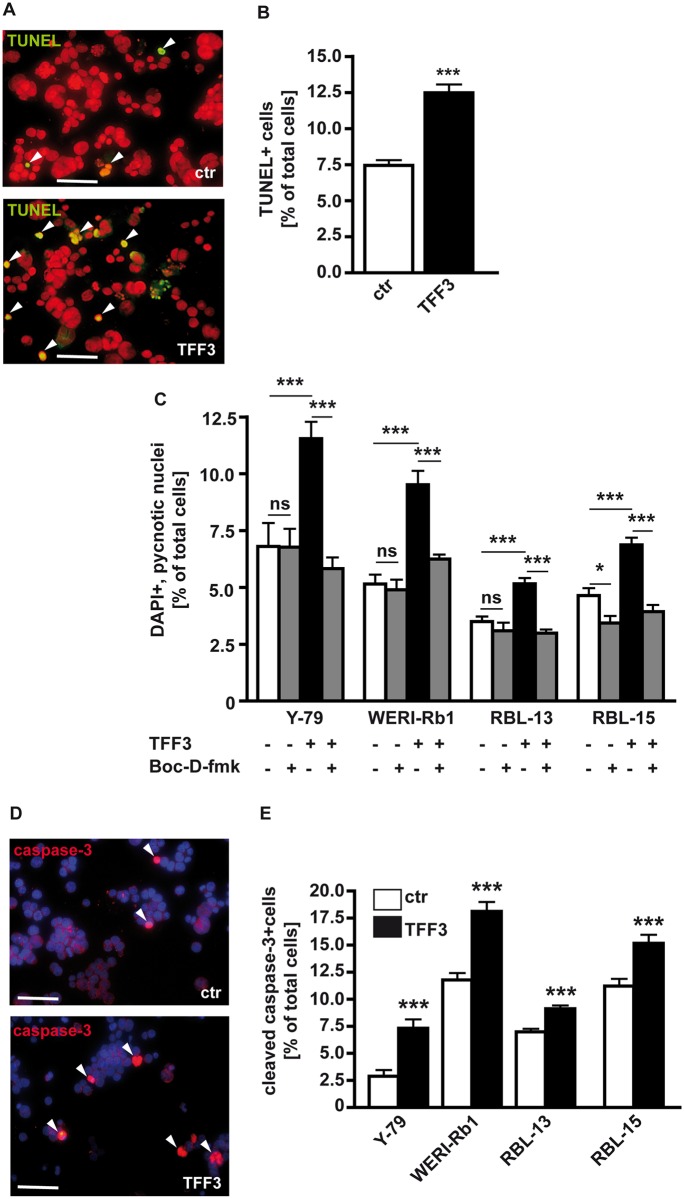
Effect of stable, lentiviral *TFF3* overexpression on apoptosis levels in retinoblastoma cell lines. (A,B) TUNEL staining of Y-79 cells and its quantification. (C) Quantification of DAPI stains in different RB cell lines after caspase blockage with Boc-D-fmk, a broad spectrum caspase inhibitor, revealed that the pro-apoptotic effect of *TFF3* overexpression is at least partially caspase dependent. (D) Representative figure of an immunocytochemical staining for active caspase-3 staining in Y-79 cells. (E) Quantification of cleaved caspase-3-positive cells revealing the involvement of caspase-3 in TFF3 mediated apoptosis in Y-79 RB cells. scale bars in A,D: 25 μm. Values are means from 3 independent experiments ± SEM. *P < 0.05; **P < 0.01; ***P < 0.001; ns = no statistical differences compared to the control group calculated by Student`s *t*-test or one-way ANOVA and Newman-Keuls Post test.

Stable *TFF3* overexpression in different RB cell lines significantly increased apoptosis levels as revealed by TUNEL assays (Fig 3A and 3B) and counts of pycnotic nuclei from DAPI stains (Fig 3C). Blockage experiments using Boc-D-fmk, a broad-spectrum caspase inhibitor, revealed the involvement of caspases in TFF3 induced apoptosis in retinoblastoma cells. In control cells, transduced with lentiviral control particles, apoptosis seems to be mostly caspase independent since the number of pycnotic nuclei was not significantly reduced by Boc-D-fmk treatment except for the cell line RBL-15, derived from a bilateral tumor, in which control cell apoptosis seems to be at least partially caspase-dependent. By contrast, forced *TFF3* expression induced a highly significant decrease in the number of pycnotic nuclei upon addition of the caspase inhibitor in all RB cell lines investigated (Fig 3C). The involvement of caspases was confirmed immunocytochemically using an antibody detecting the active form of executioner caspase-3 (Fig 3D). Cell counts from caspase-3 stains revealed that stable *TFF3* overexpression results in a significant increase in the number of cleaved and thereby activated caspase-3-positive cells in all RB cell lines analyzed (Fig 3E).

### Stable *TFF3* overexpression effects anchorage independent growth of WERI-Rb1 retinoblastoma cells

**Fig 4:**
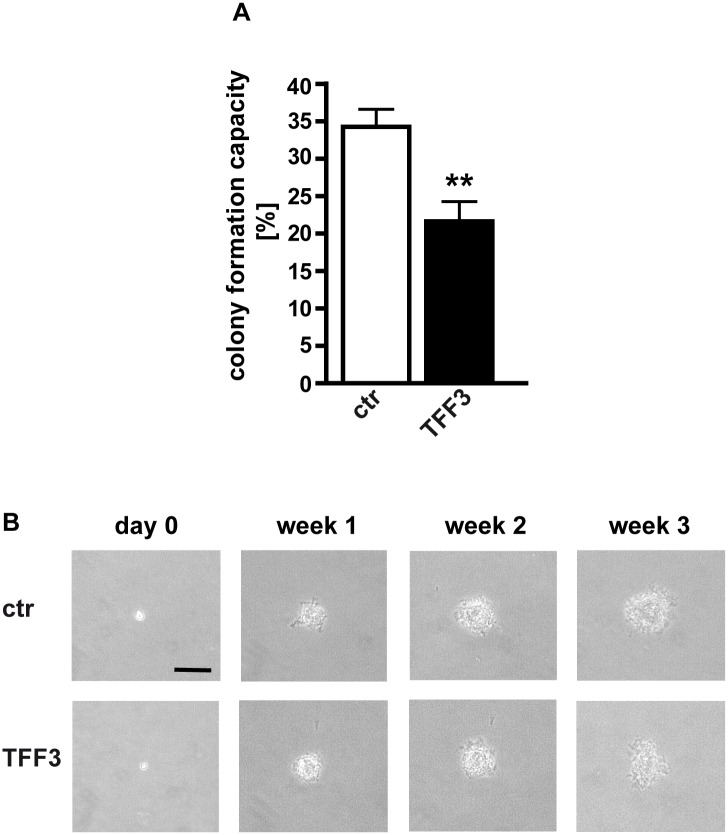
Effect of stable *TFF3* overexpression on RB cell colony formation capacity. (A) Quantification of soft agarose assays showing a significant lower capacity of TFF3 overexpressing WERI-Rb1 cells to form colonies. (B) Photographs taken from soft agarose colonies at the day of seeding (day 0), 1, 2 and 3 weeks in culture revealing that *TFF3* overexpressing WERI-Rb1 cells (TFF3) show the tendency to form smaller colonies than control cells (ctr). Scale bar: 50 μm; applies to all photographs. Values are means from 3 independent experiments ± SEM. **P < 0.01 compared to the control group calculated by Student`s *t*-test.

Forced TFF3 expression did not significantly change anchorage independent growth of Y-79 and RBL-15cells as reflected by their colony formation capacity in soft agarose (Figure B in S1 Fig). In *TFF3* overexpressing RBL-13 cells the tendency towards a reduction in colony formation capacity in soft agarose assays did not reach significance (Figure B in S1 Fig). By contrast, WERI-Rb1 cells, forming the biggest colonies among the RB cell lines analyzed by our lab [5], exhibited a significantly lower capacity to form colonies upon forced *TFF3* expression (Fig 4A). Besides, *TFF3* overexpressing WERI-Rb1 cells formed smaller colonies than control cells (Fig 4B).

### Stable *TFF3* overexpression significantly reduces the size of tumors developing from retinoblastoma cell lines

**Fig 5:**
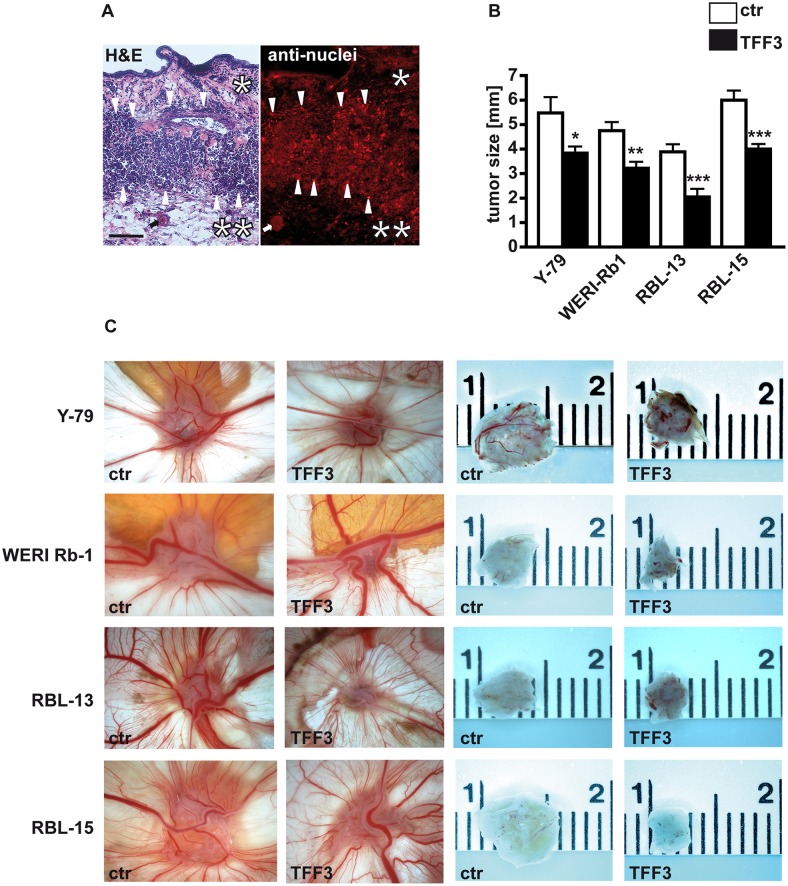
Effects of lentiviral *TFF3* overexpression on tumor formation capacity of different RB cell lines. (A) H&E stained cryosection of a tumor (left figure) that developed at the border between CAM ectoderm (*) and mesoderm (**) 7 days after grafting Y-79 RB cells on the upper CAM. Staining with a human-specific anti-nuclei antibody (red fluorescence; right figure) clearly proved the human RB cell origin of the tumor. arrows: blood vessel in CAM mesoderm; arrowheads: outline of tumor front. (B) Quantification of CAM assays. (C) Photographs of tumors *in situ* (left double column) and of ruler measurements (in cm) of excised tumors (right double column) revealing that the size of tumors developing in the upper CAM after grafting *TFF3* overexpressing RB cells (TFF3) is significantly smaller compared to those arising from control cells (ctr). scale bar: 50 μm (A; applies to both figures). Values are means from at least 3 independent experiments ± SEM. *P < 0.05; **P < 0.01; ***P < 0.001 statistical differences compared to the control group calculated by Student`s *t*-test.

H&E stained cryosection of tumors developing in the chicken CAM revealed that Y-79, WERI-Rb1, RBL-13 and RBL-15 cells grafted on the upper CAM invaded the ectoderm and formed tumors in the mesoderm or at the border between ectoderm and mesoderm (Fig 5A). Staining with a human-specific anti-nuclei antibody clearly proved that the tumors derived from human RB cells (Fig 5A). Measurements (Fig 5B) and photo-documentation of the tumors developing from Y-79, WERI-Rb1, RBL-13 and RBL-15 cells grafted on the upper CAM (Fig 5C) revealed that *TFF3* overexpressing RB cells develop significantly smaller tumors than control cells. Tumor formation capacity, however, seemed not to change significantly seven days after grafting (Figure C in S1 Fig).

**Fig 6:**
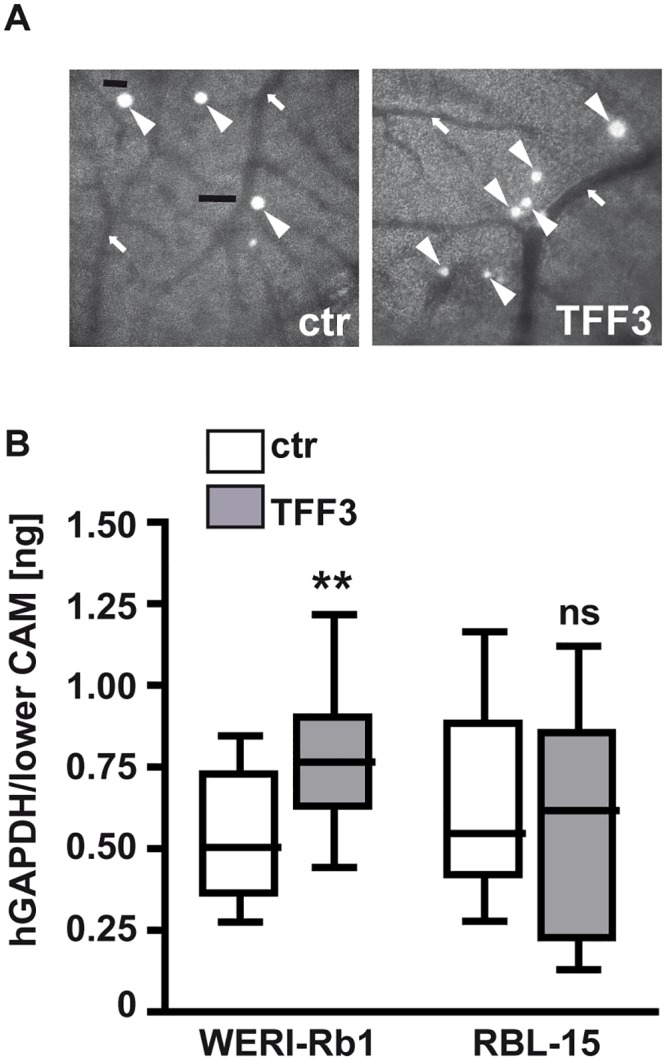
Effects of forced *TFF3* expression on RB cell migration. (A) Grayscale pictures (100x) depicting GFP-labeled WERI-Rb1 cells (arrowheads), which exited the vasculature (arrows) after injection into a CAM vein. (B) Quantification of hGAPDH content (normalized against *18S* RNA) in lower CAM punches 7 days after intravenous injection of TFF3 overexpressing (TFF3) and control (ctr) WERI-Rb1 and RBL-15 cells. Values are means from at least 3 independent experiments ± SEM.; **P < 0.01; ns = no statistical differences compared to the control group calculated by Student`s *t*-test.

After injection of *TFF3* overexpressing and control RB cells into the CAM vein, Real-time PCR analyses of lower CAM punches revealed no changes in the migration potential of RBL-15 cells after forced *TFF3* expression (Fig 6). As for RBL-13 and Y-79 cells, no GFP-positive cells were detectable in lower CAM punches, indicating that the number of migrating cells were below detection limit (data not shown). *TFF3* overexpressing WERI-Rb1 cells, by contrast, frequently exited the CAM vasculature (Fig 6A) and displayed the tendency towards a significantly higher migration rate compared to their controls (Fig 6B).

## Discussion

TFFs are known to play a key role in the maintenance and protection of epithelial and particularly mucosal surface integrity, being crucial for their restitution (reviewed in refs. [2; 14]). Experimental and clinical studies, however, indicate that TFFs are involved in more processes than just epithelial restitution, e.g. in oncogenic transformation, tumor growth, and metastasis of common human tumors (reviewed in refs. [4; 5]). TFF3 has been found to be overexpressed in various solid epithelial cancers, where it promotes cell proliferation, survival but also invasion and migration. Along this line, *TFF3* overexpression is frequently observed in gastric, pancreatic, hepatocellular, colon and breast cancer (reviewed in ref. [5]). By contrast, a recent study demonstrated that TFF3 is downregulated in most thyroid tumors [31]. Along this line our study revealed, that compared to the healthy human retina *TFF3* mRNA (data not shown) and TFF3 protein expression levels are likewise below detection levels in primary retinoblastoma samples (S2 Fig). Also RB cell lines, established from patients`primary tumors, exhibit only trace amounts of *TFF3*, and most recently, we demonstrated that the expression of *TFF3* in retinoblastoma cell lines is epigenetically down-regulated [12]. In our study presented, TFF3 executes an anti-proliferative and pro-apoptotic effect, resembling the features of a tumor suppressor, and thus, one might hypothesize that loss of TFF3 expression might contribute to retinoblastoma tumorigenesis.

In most systems studied, including the well investigated gastrointestinal tract and the ocular surface [32; 33], TFFs show protective, anti-apoptotic effects. Chen et al. (2000) describe TFF3 as an anti-apoptotic peptide secreted by intestinal goblet cells [20] and it has been demonstrated that apoptosis of enterocytes is increased in *Tff3*-deficient mice [34]. Besides, exogenous TFF3 protects human colon carcinoma cells and rat intestinal epithelial cells from apoptosis [19]. It has, however, been shown that TFF3 also interacts with peptides contributing to apoptosis [35]. Moreover, in the murine retina, our group demonstrated that recombinant TFF2 –being the only TFF peptide expressed—exerts a strong pro-apoptotic effect [17]. In the study presented, *TFF3* overexpression in Y-79, WERI-Rb1, RBL-13 and RB-15 RB cells likewise significantly induced apoptosis. In accordance with our results, TFF3 also enhances apoptosis in human articular cartilage chondrocytes [36]. In contrast to our results, transfection of colon cancer cells with *TFF3* enhanced their ability to block apoptosis [37] and in MCF-7 human mammary carcinoma cells forced expression of *TFF3* reduced apoptosis [38].

It has been shown that the cytoprotective, anti-apoptotic effect of TFF1 in gastrointestinal cells is mediated via a decrease of caspase activities [39]. For the murine retina our group was able to demonstrate that the pro-apoptotic effect of TFF2 is likewise caspase-dependent [17]. In line with these results, the present study revealed that the pro-apoptotic effect of TFF3 overexpression is caspase-dependent as well. Thus, depending on the system studied TFFs obviously exert anti- as well as pro-apoptotic effects, but in both cases caspases seem to be involved.

Microarray analysis revealed that TFF3 stimulation induces changes in the expression of genes functionally related to cell growth and proliferation [40]. In the study presented, neither the stimulation with recombinant TFF3 nor transient *TFF3* overexpression had any discernible effect on the number of proliferating cells. This can be explained in terms of the long doubling times of these cells [24] as compared to other commonly used cell lines, e. g. colon cancer cell lines. Accordingly, stable *TFF3* overexpression, allowing for long-term studies, significantly reduced cell proliferation and growth rates of all RB cell lines analyzed. In accordance with our results, TFF3 has been shown to suppress the growth of colon and colorectal carcinoma cells [41; 18]. In human corneal epithelia cells, proliferation likewise decreased 24 h after stimulation with recombinant TFF3 [42]. Along this line, forced expression of *TFF3* significantly reduced proliferation of anaplastic thyroid cancer cells [31]. By contrast, Sun et al. (2014) demonstrated that TFF3 promotes proliferation of gastric mucosal epithelial cells [43] and most recent studies likewise reported that rTFF3 enhanced the proliferation of gastric endothelial and human corneal epithelial cells [44; 45]. Moreover, forced expression of *TFF3* in mammary carcinoma and prostate cancer cells significantly increased cell proliferation, viability and survival [38; 46]. Thus, TFF3 effects on cell proliferation likewise seem to be cell type-dependent.

Our soft agarose assays testing for RB tumor cells`capacity for anchorage independent cell growth and thereby indicating their tumorigenic potential, revealed that in WERI-Rb1 cells, exhibiting the highest endogenous colony formation capacity [5], stable *TFF3* overexpression caused a significant reduction in the capacity of the cells to form colonies. By contrast, forced expression of *TFF3* in prostate cancer and mammary carcinoma cells enhances anchorage-independent growth and 3-D colony formation [38; 46]. Again, TFF3`s impact seems to be dependent on the system studied.

All TFF peptides have been shown to be motogens, enhancing the migration of cells in different systems, e.g. in response to epithelial injury[47] (for review see: [48; 49; 4; 5]). TFF3 exerts a pro-migratory effect, e.g. on corneal, bronchial, gastric mucosal epithelial, endothelial, and colorectal carcinoma cells as well as oral keratinocytes and rat fibroblasts (reviewed in ref. [5]). In the study presented, we did not detect any effects of forced *TFF3* expression on the migration potential of RBL-15 cells, derived from a bilateral tumor. The migration rate of Y-79 and RBL-13 cells, derived from unilateral primary tumors, was below the detection limit of our CAM assay independent of their TFF3 expression status. Surprisingly, *TFF3* overexpressing WERI-Rb1 cells, considered to be a less tumorigenic and aggressive RB cell line compared to Y-79 cells [6; 50; 51] and exhibiting the highest endogenous migration rates [5], displayed a significant tendency towards an increased migratory potential compared to their controls. Along this line, rTFF3 has been shown to increase the migration of human corneal epithelial cells [45]. Interestingly, this effect was dose-dependent and the effect seen with lower concentrations (10 μg/ml and 30μg/ml rTFF3) decreased with increasing concentrations (100 μg/ml and 1 mg/ml rTFF3) [45]. Forced *TFF3* expression in mammary carcinoma and prostate cancer cells likewise promoted cell migration and invasion [38; 46; 50; 52]. Transfecting *TFF3* into non-aggressive rat colorectal cancer cells not only enhanced their ability to migrate, but also to invade and behave more aggressively [37]. WERI-Rb1 cells, by contrast, did not display a higher, but a significantly lower capacity for anchorage independent cell growth, indicating that their tumorigenic potential did not increase.

It has been shown that forced expression of *TFF3* in mammary carcinoma cells increased tumor size [38; 46], contradicting our findings that *TFF3* overexpression reduced the size of tumors forming from RB cells grafted on the CAM. Although in our study presented all tumors developing from RB cells inoculated on the upper CAM appeared highly vascularized and we did not detect any signs of necrosis in sections of excised tumors, one cannot completely rule out the possibility that the CAM model might not provide entirely sufficient angiogenesis conditions for an extensively fast tumor growth and thus, tumors potentially do not develop any further but decrease in size. Alternatively, these contradictory data again can be explained in terms of cell-specific actions: in mammary carcinoma and prostate cancer cells forced expression of *TFF3* significantly increased cell proliferation, viability and survival [38; 46], whereas it triggered a strong pro-apoptotic and anti-proliferative effect in retinoblastoma cell lines investigated in our study. An anti-proliferative effect of forced *TFF3* expression has also been observed in anaplastic thyroid cancer cells in a recent study by Abols et al. (2015) and the authors suggested that TFF3 may act as a tumor suppressor or an oncogene depending on the cellular context [31].

Summarizing our data show that in RB cell lines a significant pro-apoptotic, anti-proliferative, and tumor suppressive effect can be ascribed to TFF3, setting a new starting point for future additive chemotherapeutic approaches to reduce retinoblastoma tumor size. In this context, one must, however, be aware of a potential pro-migratory effect of TFF3 in some tumor entities and thus routinely follow up on the formation of metastases.

## Supporting Information

S1 Fig Confirmation of stable, lentiviral *TFF3* overexpression by Real-time PCR (Figure A), effect of stable *TFF3* overexpression on colony formation capacity (Figure B), and effect of forced TFF3 expression on tumor formation capacity of different RB cell lines (Figure C). Values are means from at least 3 independent experiments ± SEM. *P < 0.05; ns = no statistical differences compared to the control group calculated by Student`s *t*-test.
(TIF)

S2 Fig Comparison of TFF3 expression in a healthy human retina and retinoblastoma sample as revealed by immunohistochemistry using DAB detection (brown signal) and haematoxylin counterstaining (blue nuclei staining). (A) Paraffin section of a healthy human retina incubated without primary TFF3 antibody serving as a negative control (neg. ctr.). (B) Immunohistochemical detection of TFF3 expression (TFF3) in a paraffin section of a healthy human retina. Arrowheads indicate TFF3-positive cells in the ganglion cell layer (GCL) and the inner nuclear layer (INL). Besides, the inner and outer segments of photoreceptors stained positively for TFF3. scale bar in A: 20 μm (also applies to B,G,H and insets in C,D). (C) Section of a retinoblastoma sample incubated without primary TFF3 antibody serving as a negative control. (D) Section of a retinoblastoma sample stained with a specific TFF3 antibody. Insets depict the region of the outer nuclear layer (ONL) and INL at higher magnification, both exhibiting no positive staining for TFF3. TM: tumor mass. scale bar in C: 50 μm (also applies to D-F). (E, F) Overview of the retinoblastoma tumor mass. (G,H) Close up of the retinoblastoma tumor mass depicted in E and F. No TFF3 signal was detectable in the tumor mass.
(TIF)
