# Supplementary figures and images for: Forced Trefoil Factor Family Peptide 3 (TFF3) Expression Reduces Growth, Viability, and Tumorigenicity of Human Retinoblastoma Cell Lines
Source: PLoS One. 2016 Sep 14;11(9):e0163025. doi: 10.1371/journal.pone.0163025 (PMC5023179; doi:10.1371/journal.pone.0163025)

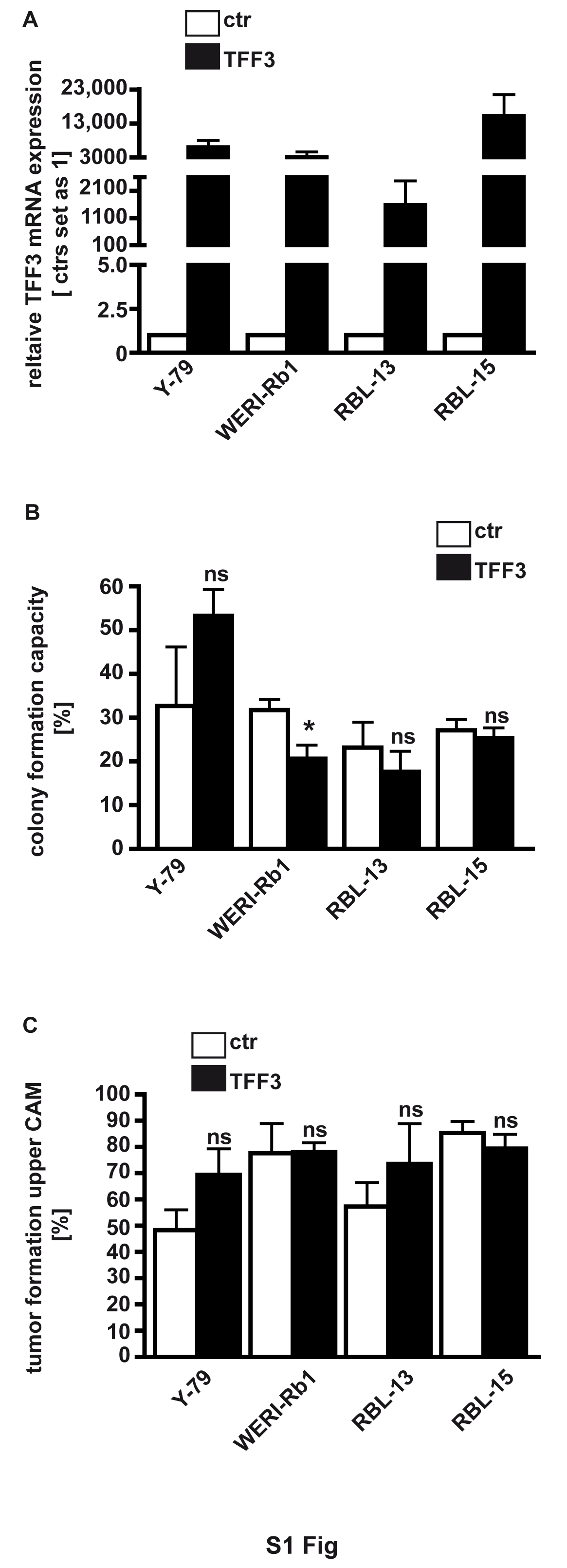

Supplement: S1 Fig — Values are means from at least 3 independent experiments ± SEM. *P < 0.05; ns = no statistical differences compared to the control group calculated by Student`s t-test. (TIF) [file pone.0163025.s001.tif]

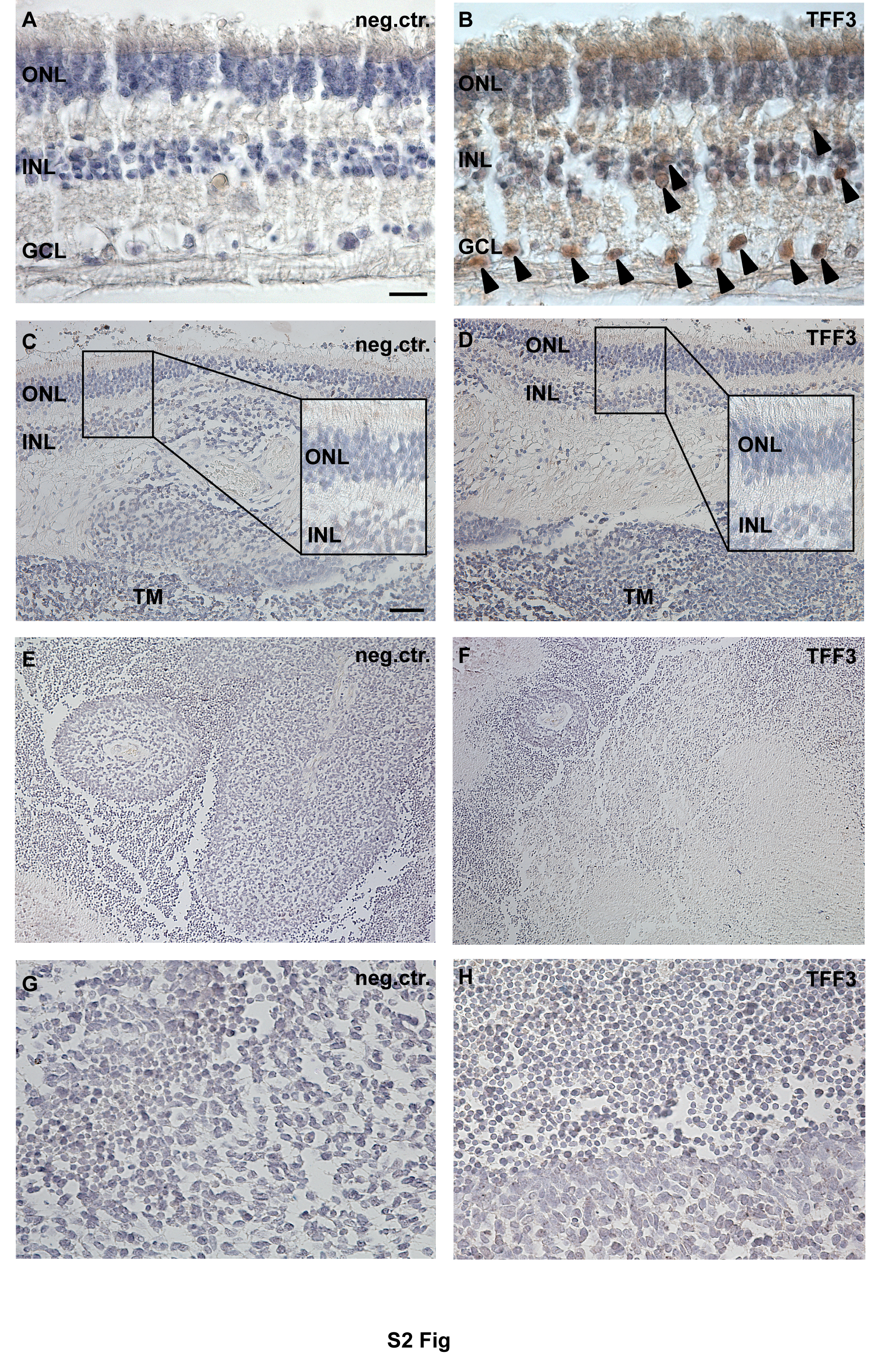

Supplement: S2 Fig — (A) Paraffin section of a healthy human retina incubated without primary TFF3 antibody serving as a negative control (neg. ctr.). (B) Immunohistochemical detection of TFF3 expression (TFF3) in a paraffin section of a healthy human retina. Arrowheads indicate TFF3-positive cells in the ganglion cell layer (GCL) and the inner nuclear layer (INL). Besides, the inner and outer segments of photoreceptors stained positively for TFF3. scale bar in A: 20 μm (also applies to B,G,H and insets in C,D). (C) Section of a retinoblastoma sample incubated without primary TFF3 antibody serving as a negative control. (D) Section of a retinoblastoma sample stained with a specific TFF3 antibody. Insets depict the region of the outer nuclear layer (ONL) and INL at higher magnification, both exhibiting no positive staining for TFF3. TM: tumor mass. scale bar in C: 50 μm (also applies to D-F). (E, F) Overview of the retinoblastoma tumor mass. (G,H) Close up of the retinoblastoma tumor mass depicted in E and F. No TFF3 signal was detectable in the tumor mass. (TIF) [file pone.0163025.s002.tif]
